# Draft genome sequences of 12 *Vibrio* Harveyi clade isolates from Hong Kong coastal waters

**DOI:** 10.1128/mra.00231-25

**Published:** 2025-05-19

**Authors:** A. J. Hu, A. N. H. Cheung, K. M. Leung, G. K. K. Lai, S. D. J. Griffin

**Affiliations:** 1 Shuyuan Molecular Biology Laboratory, The Independent Schools Foundation Academy, Hong Kong SAR, China; Portland State University, Portland, Oregon, USA

**Keywords:** *Vibrio harveyi*, Harveyi clade, Hong Kong, coastal waters

## Abstract

Twelve *Vibrio* Harveyi clade isolates were obtained from Hong Kong coastal waters. Their draft genome sequences were assembled and found to comprise *V. harveyi* (×2), *Vibrio owensii* (×5), *Vibrio campbellii* (×2), and *Vibrio parahaemolyticus* (×3), with estimated genome sizes 4.9–6.2 Mbp (44.5%–45.8% G + C).

## ANNOUNCEMENT

The Gram-negative, motile marine bacteria, *Vibrio harveyi*, *Vibrio owensii*, *Vibrio campbellii*, and *Vibrio parahaemolyticus* are core members of the Harveyi clade (1) and commonly found in surface waters and sediments (2) and also marine debris (3). Though host-associated and sometimes commensal, they are frequently mutualistic bioluminescent symbionts (4). Nevertheless, their excessive environmental abundance is a broad indicator of pollution (5), and virulent strains are responsible for diseases affecting aquaculture and corals (6, 7), as well as acute gastroenteritis in humans (2). The 12 free-living strains introduced here were isolated from water and sediment samples from Hong Kong coastal waters (see Table 1) as part of a broad survey of cross-species gene transfer.

**TABLE 1:** Sampling, sequencing, and genomic analysis data for twelve Harveyi clade isolates

Strain	ANHC.C1D	TX.TDP18	TX.T19	TX.T17	TX.TSW12	TX.TDP14	AYW.12	TX.TDP6	TX.T18	DVW.05	DVW.10	ANHC.C2L
Species	*V. harveyi*	*V. harveyi*	*V. owensii*	*V. owensii*	*V. owensii*	*V. owensii*	*V. owensii*	*V. campbellii*	*V. campbellii*	*V. para-haemolyticus*	*V. para-haemolyticus*	*V. para-haemolyticus*
Genbank accession	JBLWED000000000	JBLWDW000000000	JBLWEB000000000	JBLWDX000000000	JBLWDY000000000	JBLWDV000000000	JBLWEC000000000	JBLWDZ000000000	JBLWEA000000000	JBLWDT000000000	JBLWDU000000000	JBLWEE000000000
est. genome size (Mbp)^*a*^	5.68	5.68	5.85	5.90	5.95	6.18	5.81	5.66	5.16	5.26	5.69	4.92
% G + C	44.96	45.04	45.63	45.59	45.59	45.62	45.77	45.32	44.52	45.64	45.31	45.37
*Sampling*												
Biosample accession	SAMN46984444	SAMN46982965	SAMN46984320	SAMN46983008	SAMN46983018	SAMN46982774	SAMN46984327	SAMN46983020	SAMN46984318	SAMN46982718	SAMN46982723	SAMN46984514
Sampling date	2023-09-17	2022-05-23	2022-05-23	2022-05-23	2022-05-23	2022-05-23	2021-07-05	2022-05-23	2022-05-23	2021-04-13	2021-04-13	2023-09-17
GPS	22.286017, 114.162247	22.244551, 114.187313	22.237610, 114.188239	22.237610, 114.188239	22.220735, 114.214343	22.244551, 114.187313	22.247517, 114.150967	22.244551, 114.187313	22.237610, 114.188239	22.283347, 114.184740	22.283347, 114.184740	22.286017, 114.162247
Location	Central Ferry Pier No.9	Deepwater Bay	Island Road	Island Road	Stanley	Deepwater Bay	Aberdeen Typhoon Shelter	Deepwater Bay	Island Road	Causeway Bay Typhoon Shelter	Causeway Bay Typhoon Shelter	Central Ferry Pier No.9
Sample type	Surface seawater	Surface seawater	Surface seawater	Surface seawater	Surface seawater	Surface seawater	Sediment	Surface seawater	Surface seawater	Seawater depth 0.5 m	Seawater depth 1.0 m	Surface seawater
Appearance of colonies on ChromAgar Vibrio	Pale purple	Purple	Blue	Blue	White	Purple-blue	Blue	Purple	Blue	White	White	White (purple tinge)
*Sequencing*												
Sequencing platform	NovaSeq	Miseq	Miseq	Miseq	Miseq	Miseq	Miseq	Miseq	Miseq	Miseq	Miseq	NovaSeq
SRA accession	SRX27838615	SRX27838607	SRX27838359	SRX27838403	SRX27838405	SRX27837604	SRX27837616	SRX27837626	SRX27837620	SRX27837460	SRX27837472	SRX27838619
# raw reads	8,739,546	1,762,840	2,244,022	2,034,566	1,863,654	1,729,116	949,460	2,250,898	2,158,530	760,268	1,747,670	9,087,148
Mean read 3length (bp)	250	231	216	232	235	233	205	222	203	236	176	250
Total (Mbp)	2,185	407.2	484.7	472.0	438.0	402.9	194.6	499.7	438.2	179.4	307.6	2272
Mean read coverage	352	72	83	81	74	65	34	88	85	34	54	447
*Assembly*												
# contigs	80	151	130	108	111	264	331	365	111	472	451	64
N50	649,009	66,985	91,572	113,681	118,285	60,209	32,098	42,073	98,912	24,154	22,665	388,288
Completeness %^*b*^	99.38	98.50	99.34	99.40	100	100	99.74	99.87	99.34	97.00	95.52	98.72
Contamination %^*b*^	4.18	4.85	1.37	1.37	1.16	1.29	0.85	0.09	0.48	4.16	4.59	1.03
*Analysis*												
# CDS (coding)^*c*^	5,064	5,104	5,160	5,132	5,246	5,470	5,161	5,017	4,535	4,770	5,254	4,386
rRNA genes (5S + 16S + 23S)^*c*^	1, 1, 3	1, 1, 1	1, 1, 3	1, 1, 3	–, 2, 4	–, 3, 2	1, 3, 3	2, 3, 3	2, 1, 3	1, 3, 2	1, 2, 2	3, 3, 4
tRNA genes^*c*^	102	94	104	103	96	98	79	98	78	81	66	94
*luxR*	1	1	1	1	1	1	1	1	1	1	1	1
*lukAB*	0	0	0	0	0	0	0	1	1	1	1	1
*avrD*	0	0	1	1	2	1	1	0	0	0	0	0
*toxR*	0	0	1	1	1	1	1	1	1	1	1	1
*bla*_CARB-18_	0	0	0	0	0	0	0	0	0	1	1	1

^*a*^ By RASTtk (8).

^*b*^ By CheckM v1.2.3 (9).

^*c*^ By PGAP v6.9 (10).

Sediment (1 g) was vortexed (20 s) in 3.5% saline (19 mL) and allowed to settle (5 min) before a 100 µL aliquot of the clearer supernatant was spread onto CHROMagar Vibrio agar (CHROMagar, Paris, France; 100 µL aliquots of seawater samples were spread without dilution). Following overnight incubation, selected colonies were purified by single-colony streaking (≥5 times) on Luria agar (11). For DNA extraction, a single colony was streaked on this medium to harvest overnight growth from the plate (DNeasy PowerSoil Pro kit, Qiagen GmbH, Hilden, Germany). All incubations were at 27°C for 24 hours.

UV absorbance (BioDrop µLITE, Biochrom Ltd., UK) and PicoGreen assays (12) were used for DNA quality/quantity evaluations before using Illumina MiSeq (v3 chemistry) or Illumina NovaSeq 6000 platforms to sequence paired-end short-read sequencing libraries, prepared and barcoded using, respectively, the NexteraXT DNA Library Preparation Kit or NovaSeqX Series 10B Reagent Kit (Illumina Inc., USA). Reads were quality-filtered and trimmed using TrimGalore! v0.6.7 (https://github.com/FelixKrueger/TrimGalore; stringency:3; -e:0.2) and assembled by Unicycler v.0.5.0 (9). Default parameters were used for all software unless otherwise specified. Sequencing data and analysis for all 12 isolates, including CheckM v1.2.3 metrics for completeness/contamination (8), are given in Table 1.

Genomes were annotated by RASTtk v1.9.1 in BV-BRC v3.46.3 (10) and by NCBI PGAP v6.9 (13). NCBI BLAST and JSpeciesWS v4.2.1 (14, 15), as well as a codon tree generated in BV-BRC by RAxML v8 (16), were used for species identification, thus classifying the twelve strains as *V. harveyi* (×2), *V. owensii* (×5), *V. campbellii* (×2), and *V. parahaemolyticus* (×3). All strains carry the luminescence operon transcriptional activator *luxR*, a Harveyi clade-specific virulence factor (17), as well as genes encoding the arsenite efflux transporter, *acr3* (18), and the *tupABC-mobAB* tungstate-uptake system (19, 20). The species-linked distribution of the leukocidin family pore-forming toxin *lukAB* and of *avrD* (21), the cholera toxin transcriptional activator *toxR* (22), and class A beta-lactamase *bla*CARB-18 (23) is shown in Table 1.

## Data Availability

Hyperlinked accession numbers for all 12 isolates are given in Table 1.
